# Silencing Formin-like 2 inhibits growth and metastasis of gastric cancer cells through suppressing internalization of integrins

**DOI:** 10.1186/s12935-018-0576-1

**Published:** 2018-06-01

**Authors:** Banghua Zhong, Kewei Wang, Hao Xu, Fanmin Kong

**Affiliations:** Department of Gastric, Intestine and Hernia Surgery, The First Affiliated Hospital of China Medical University, Shenyang, 110001 People’s Republic of China

**Keywords:** Apoptosis, Formin-like 2, Gastric cancer, Integrin internalization, Invasion, Migration, Proliferation

## Abstract

**Background:**

Formin-like 2 (FMNL2) is a member of Formin family which governs cytokinesis, cellular polarity and morphogenesis. Dysregulation of FMNL2 has been discovered in cancers and is closely related to cancers. However, the role of FMNL2 in gastric cancer remains unclear. In this study, we aimed to investigate the role of FMNL2 in gastric cancer cells.

**Methods:**

A FMNL2-specific shRNA was employed to decrease the endogenous expression of FMNL2. Then the degree of proliferation, apoptosis, migration and invasion of gastric cancer cells was assessed by MTT assay, flow cytometry, wound healing assay and transwell assay, respectively. The expression and distribution of FMNL2 and protein kinase C (PKC) α was detected by immunofluorescence. The internalization of integrins was detected by enzyme-linked immunosorbent assay.

**Results:**

Our results showed that silencing FMNL2 suppressed proliferation, migration and invasion, and induced apoptosis of gastric cancer cells. The integrin internalization induced by PKC was declined by FMNL2 silencing.

**Conclusions:**

Our study reveals that silencing FMNL2 suppresses growth and metastasis of gastric cancer cells. Modulation on integrin internalization may be implicated in the role of FMNL2 in growth and migration of gastric cancer cells. Our study indicates that FMNL2 may become a potential therapeutic target for gastric cancer.

## Background

Gastric cancer, which threatens human health, is the fifth most common cancer worldwide with approximately one million cases in 2012 [1]. Surgery is the curative modality for gastric cancer treatment. However, the recurrence rate is still high, and the 5-year survival rate is low [2].

Integrins are a group of αβ heterodimers. Integrins are parts of the major receptors spanning through the lipid bilayer of cells. Integrins bind directly to extracellular matrix components, transduce signals outside-in and activate intracellular signals in context of cytokine receptors or growth factor receptors. Internalization and recycling of integrins emerges as a major player in controlling integrin action [3]. Integrins also contribute to tumor progression and are implicated in cell survival, metastasis, angiogenesis and drug resistance [4–8]. They also influence the response of host cells to cancers [8]. Integrins are regarded as appealing targets for tumor therapy [8].

Formins are a group of Rho guanosine triphosphatase (GTPase) effectors, governing cytokinesis, polarity, adhesion and morphogenesis [9]. Diaphanous-related Formins (DRFs) are a conserved subgroup of Formins. They are involved in a wide range of cellular processes, including filopodium formation, cell migration and cell polarity [9–11], and contribute to many physiological or pathological processes [12–14]. Formin-like 2 (FMNL2), a member of DRFs, locates on chromosome 2q23.3. FMNL2 contains a GTPase-binding domain and an autoregulatory domain [15], and acts as both a downstream effector and an upstream modulator of Rho family GTPases [11]. FMNL2 plays a key role in actin filament nucleation or elongation which influences cell morphology [9]. FMNL2 also has a close relationship with cancer. For example, FMNL2 enhances the growth and metastasis of colon cancer in which FMNL2 is highly expressed [16–18]. Moreover, FMNL2 performs an inhibitory effect on motility of hepatocarcinoma which shows a lower FMNL2 level than normal livers [19]. In addition, patients with aberrant FMNL2 expression show a lower 5-year survival rate [19]. Dysregulation of FMNL2 has been discovered in several types of cancers and is associated with the development of aggressive tumors [16–20]. However, the role of FMNL2 in gastric cancer remains unclear.

In the present study, we aimed to investigate the effect of FMNL2 silencing on gastric cancer cells. The results of our study indicate that FMNL2 may act as an oncogene in gastric cancer cells and has the potential to become a therapeutic target for gastric cancer.

## Materials asnd methods

### Cells

Gastric cancer cell lines BGC-823, MGC-803, HGC-27 were obtained from Zhongqiaoxinzhou Biotechnology Co., Ltd (Shanghai, China). SGC-7901 cell line was obtained from CHI Scientific (Jiangyin, China). BGC-823 and MGC-803 cells were grown in Dulbecco’s Modified Eagle’s Medium (Gibco, Grand Island, NY, USA) with 10% fetal bovine serum (FBS; Hyclone, Logan, UT, USA). SGC-7901 and HGC-27 cells were grown in RPMI 1640 medium (Gibco) with 10% FBS. All these cells were cultured in a humid atmosphere at 37 °C with 5% CO_2_.

### Transfection

HGC-27 cells were seeded in a 6-well plate (2 × 10^5^ cells/well) and cultured in a cell incubator. Twenty-four hours later, the cells were transfected with FMNL2 shRNA (target sequence: 5′-GTGGTAGCAGGTAACTCTG-3′) or negative control using Lipofectamin 2000 Reagent (Invitrogen, Carlsbad, CA, USA) according to the manufacturer’s protocol. Then the cells were maintained in cell medium with 800 μg/ml G418 (Invitrogen) for 7 days to select stably transfected cells. Cells stably transfected with FMNL2 shRNA were named as FMNL2 silencing. Cells stably transfected with negative control were named as negative control. Cells without transfection were named as cells only.

### Quantitative real-time PCR (qRT-PCR)

Total RNA in cells from each group was extracted using a High-purity Total RNA Fast Extraction kit (BioTeke, Beijing, China) and reverse transcribed to cDNA using Oligo(dT)_15_ and Super M-MLV Reverse Transcriptase (BioTeke) according to the manufacturers’ instructions. mRNA level of FMNL2 in each group was measured by qRT-PCR (SYBR Green method) with cDNA as the template. The following primers were used: forward primer for FMNL2, 5′-CCCGCTCTGGAAGACATT-3′; reverse primer for FMNL2, 5′-CTGCCAACAGTTCTAAGACAAG-3′; forward primer for β-actin, 5′-CTTAGTTGCGTTACACCCTTTCTTG-3′; reverse primer for β-actin, 5′-CTGTCACCTTCACCGTTCCAGTTT-3′. SYBR Green Reagent was obtained from Solarbio (Beijing, China). The relative mRNA level of FMNL2 was normalized to β-actin and calculated using 2^−ΔΔCt^ method.

### Western blot

Proteins in each group were extracted using radio immunoprecipitation assay lysis buffer (Beyotime Biotechnology, Haimen, China) containing 1% phenylmethanesulfonyl fluoride (Beyotime Biotechnology). The concentration of proteins in each group was measured using a BCA Protein Assay Kit (Beyotime Biotechnology). Forth microgramme proteins in each group were separated by sodium dodecyl sulfate polyacrylamide gel electrophoresis. After electrophoresis, the proteins were transferred onto polyvinylidene fluoride membranes (Millipore, Bedford, MA, USA). The membranes were blocked with 5% skim milk, followed by incubating with primary antibodies against FMNL2 (1:500; Novus, Oakville, ON, Canada), E-cadherin (1:2000, Proteintech, Wuhan, China), Vimentin (1:2000, Proteintech), N-cadherin (1:2000, Proteintech) or β-actin (1:500; Bioss, Beijing, China) at 4 °C overnight. After rinsing in Tris buffered saline with Tween (TBST), the membranes were incubated with horseradish peroxidase (HRP)-conjugated goat-anti-rabbit IgG(H+L) or HRP-conjugated goat-anti-mouse IgG(H+L) (1:5000; Beyotime Biotechnology) at 37 °C for 45 min. After rinsing in TBST, the targeted bands were visualized using an ECL Kit (Beyotime Biotechnology).

### 3-(4,5-Dimethyl-2-thiazolyl)-2,5-diphenyl-2-H-tetrazolium bromide (MTT) assay

Cells in each group were seeded into 96-well plates (4 × 10^3^ cells/well) and cultured in a cell incubator for 0, 24, 48 and 72 h, respectively. Then MTT (Sigma, St. Louis, MO, USA) at a final concentration of 0.5 mg/ml was added into each well. After incubating for additional 4 h, 150 μl dimethyl sulfoxide (DMSO, Sigma) was added into each well to dissolve the crystals after removal of supernate. Thereafter, the absorbance at 490 nm was measured with a microplate reader (BIOTEK, Winooski, VT, USA).

### Flow cytometry

Degree of apoptosis in each group was analyzed by flow cytometry with a Cell Apoptosis Detection Kit (KeyGen, Nanjing, China). Cells in each group were collected, rinsed in phosphate buffered saline (PBS) and resuspended in 500 μl binding buffer. Then 5 μl Annexin–Fluorescein Isothiocyanate (FITC) and 5 μl Propidium Iodide (PI) were added into cells for incubation for additional 15 min at room temperature in dark. The cells were then analyzed with a flow cytometer (BD, Franklin Lakes, NJ, USA).

### Wound healing assay

Cells in each group were seeded into a 6-well plate. When the confluence reached 90%, the cell medium was changed to fresh serum-free medium. Then the cells were treated with mitomycin C (1 μg/ml; Sigma) for 1 h. Thereafter, scratches were made on single-cell surface with 200 μl pipette tips. The cells were washed with serum-free medium to remove cell debris, and allowed to migrate for 24 h in serum-free medium. Images of cells were captured with a microscope at 0 h and 24 h. The relative migrate rate was calculated using the following formula: relative migrate rate = (distance between gap at 0 h − distance between gap at 24 h)/distance between gap at 0 h × 100%.

### Transwell assay

Matrigel (BD) was thawed at 4 °C and diluted at 1:3 with serum-free medium. Transwell inserts (Corning, Tewksbury, MA, USA) were put into a 24-well plate and pre-coated with 40 μl of diluted Matrigel. Cells in each group were digested with trypsin (Sigma) and made into single-cell suspension. 200 μl single-cell suspension containing 1 × 10^4^ cells was added into the upper chamber and 800 μl of RPMI 1640 medium containing 30% FBS was added into the lower chamber. The cells were allowed to invade for 24 h. Then the cells were rinsed with PBS, fixed with 4% paraformaldehyde (Sinopharm, Shanghai, China) at room temperature for 20 min, and stained with 0.5% crystal violet dye (Amresco, Solon, OH, USA) for 5 min. Images of cells were captured under a microscope at 200× magnification.

### Immunofluorescence assay

Cells were treated with DMSO or 12-*O*-tetradecanoylphorbol-13-acetate (TPA; 200 nM; Cell Signaling Technology, Danvers, MA, USA) for 5 min. The cells were rinsed with PBS and permeabilized with 0.1% TritonX-100. After blocking with goat serum, the cells were incubated with FMNL2 antibody (1: 100; Novus) or protein kinase C (PKC) α antibody (1: 50; Proteintech, Wuhan, China) at 4 °C overnight. Thereafter, the cells were rinsed with PBS and incubated Cy3-conjugated goat-anti-rabbit IgG(H+L) or FITC-conjugated goat-anti-mouse IgG(H+L) (1:200; Beyotime Biotechnology). The cells were then rinsed with PBS and observed under a fluorescence microscope (600×; OLYMPUS, Tokyo, Japan).

### Integrin internalization assay

Cells were labeled with EZ-Link Sulfo-NHS-SS-Biotin (0.5 mg/ml; Thermo Fisher Scientific, Rockford, IL, USA) on ice for 1 h. Then the cells were treated with DMSO or TPA for 30 min. The cells were kept on ice and rinsed with ice-cold PBS. Biotin residual on cell surface was removed by incubating in Tris buffer containing 20 mM MesNa (Aladdin, Shanghai, China) at 4 °C for 15 min. Superfluous MesNa was quenched by 20 mM iodoacetamide (Aladdin). Then the cells were collected and lysed in lysis solution (containing 200 mM NaCl, 15 mM NaF, 75 mM Tris, 7.5 mM EDTA, 7.5 mM EGTA, 1.5 mM Na3VO4, and 1.5% TritonX-100). The supernatant was collected by centrifugation at 1500×*g* at 4 °C for 10 min. After measuring the concentration with a BCA Protein Assay Kit, the level of biotinylated integrin was analyzed by enzyme-linked immunosorbent assay (ELISA). Briefly, the ELISA plate was precoated with antibodies against integrin-β1 (Proteintech), integrin-α2 (Thermo Fisher Scientific), or integrin-α5 (Proteintech) at 4 °C overnight, and then blocked with 5% bovine serum albumin. Equivalent sample from each group was added into each well and incubated at 4 °C overnight. Streptavidin-conjugated HRP (Beyotime Biotechnology) was added into each well after rinsing and incubated at 4 °C for 1 h. Thereafter, the plate was rinsed, and OPD Color-Substrate Solution (Sigma) was added into each well for incubation at 37 °C for 25 min. After adding of stop solution, the absorbance was measured at 492 nm.

### Statistical analysis

All experiments were carried out in triplicate. The results were shown as mean ± SD. Differences between groups were analyzed with one-way analysis of variance. p < 0.05 was considered as significant.

## Results

### FMNL2 expression level in gastric cancer cell lines

**Fig. 1:**
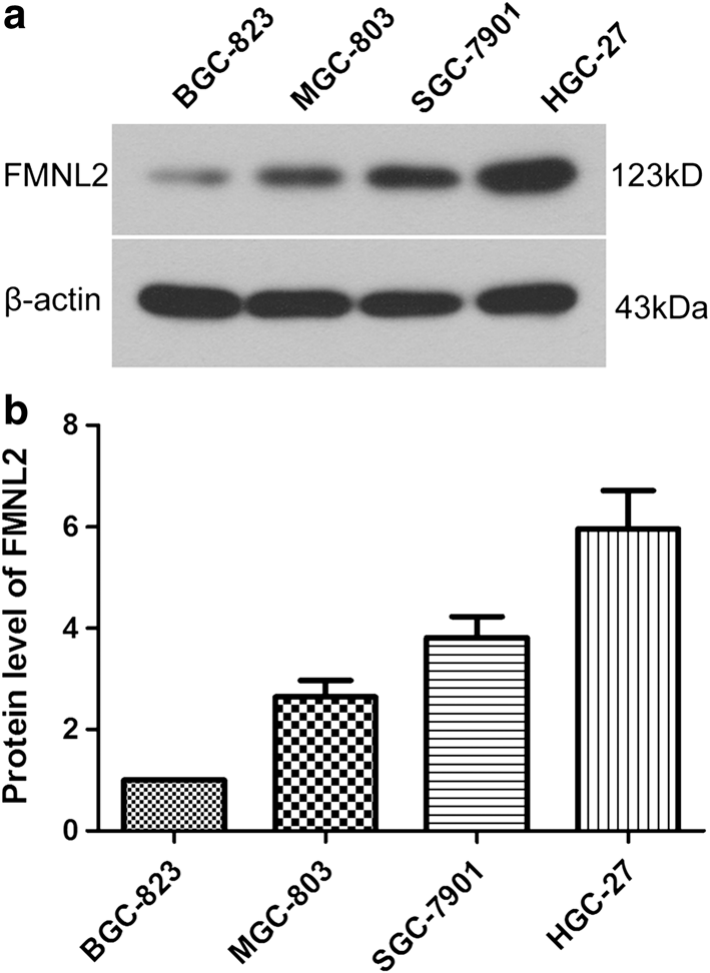
FMNL2 level in gastric cancer cell lines. **a** Protein level of FMNL2 in gastric cancer cell lines BGC-823, MGC-803, SGC-7901 and HGC-27 was detected by western blot. β-Actin served as the internal control. **b** Relative FMNL2 level in each cell line was calculated. All experiments were repeated three times. The results were shown as mean ± SD

To select a gastric cancer cell line for our study, the FMNL2 level in gastric cancer cell lines BGC-823, MGC-803, SGC-7901 and HGC-27 was detected by Western blot. As shown in Fig. 1, the FMNL2 level was 2.64 ± 0.33-fold in MGC-803 cells, 3.82 ± 0.41-fold in SGC-7901 cells, and 5.96 ± 0.75-fold in HGC-27 cells, compared with that in BGC-823 cells. Thus, HGC-27 cell line, with the highest FMNL2 expression, was selected for subsequent experiments.

### FMNL2 shRNA decreases the FMNL2 level in HGC-27

**Fig. 2:**
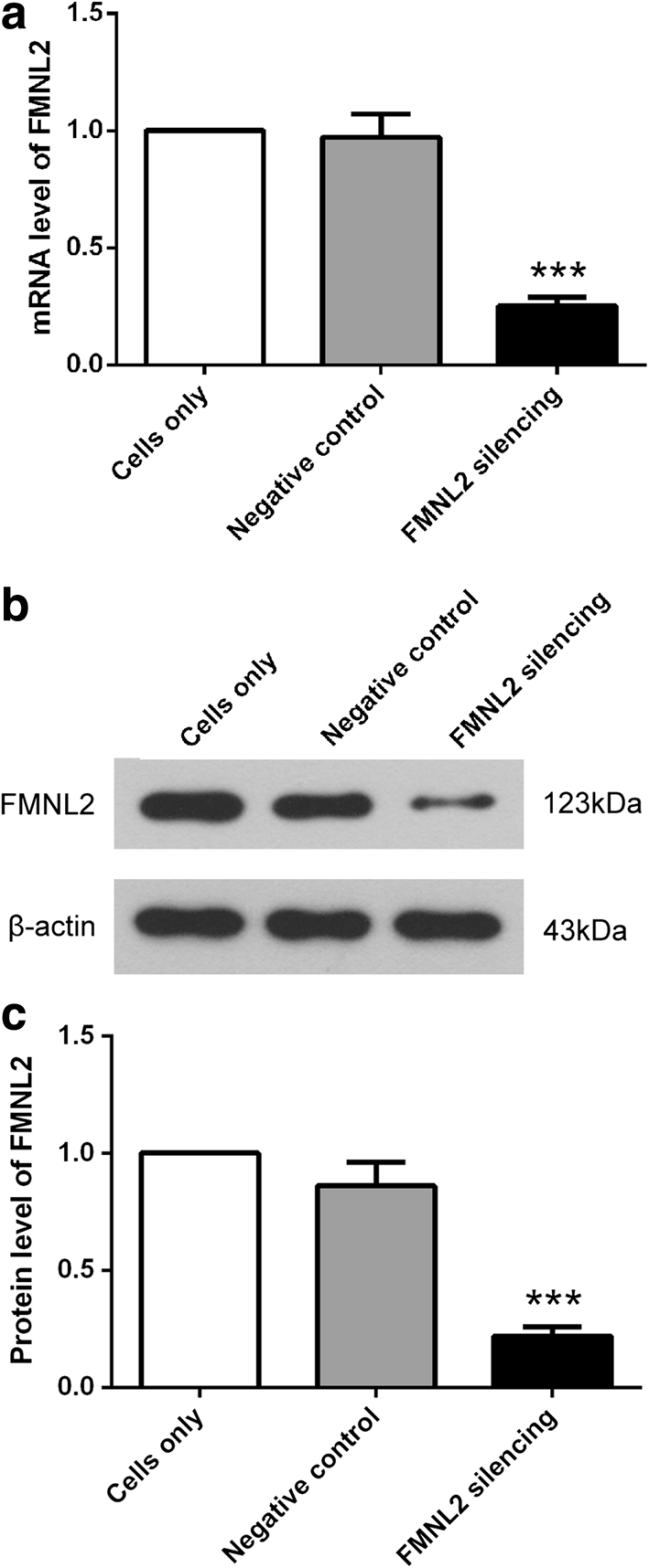
FMNL2 shRNA decreases FMNL2 level in HGC-27 cells. **a** mRNA level of FMNL2 in HGC-27 cells was measured by quantitative real-time PCR after FMNL2 silencing. mRNA level of FMNL2 was normalized to β-actin, and relative mRNA level was calculated using 2^−ΔΔCt^ method. **b**, **c** After FMNL2 silencing, protein level of FMNL2 was assessed by western blot with β-actin as internal control. Each experiment was repeated three times. The results were shown as mean ± SD. ***p < 0.001 compared with negative control cells

To explore the role of FMNL2 in gastric cancer, a FMNL2-specific shRNA was employed in our study. Then the efficiency of FMNL2 shRNA was verified by qRT-PCR and western blot. After transfection with FMNL2 shRNA, the relative mRNA level of FMNL2 was decreased to 25 ± 4% (Fig. 2a), and the relative protein level of FMNL2 was decreased to 22 ± 4% (Fig. 2b, c). These results demonstrate that FMNL2 shRNA declines FMNL2level effectively, both at mRNA level and protein level.

### FMNL2 silencing inhibits proliferation and induces apoptosis of HGC-27 cells

**Fig. 3:**
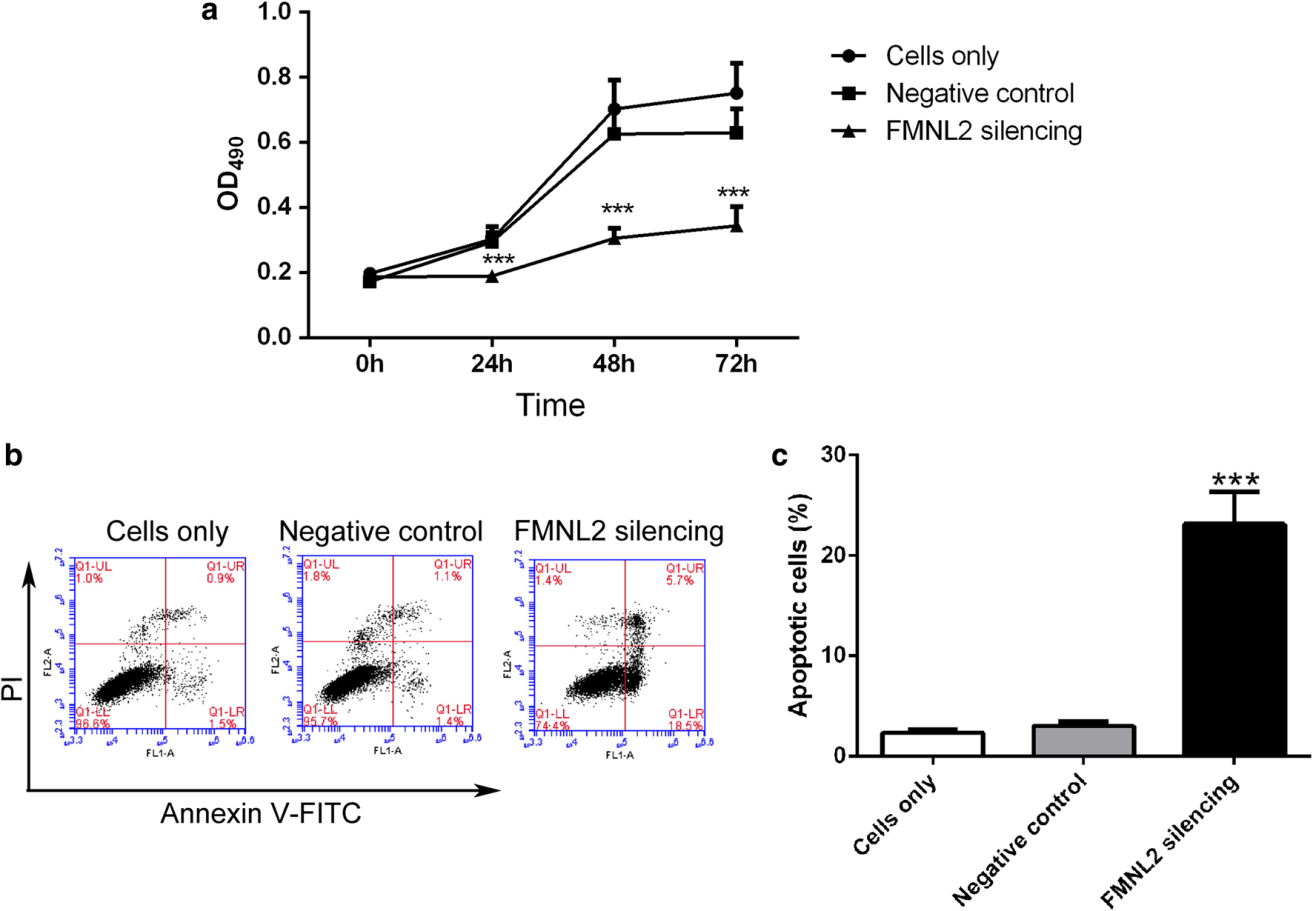
FMNL2 silencing inhibits growth of HGC-27 cells. **a** Cell viability of HGC-27 in each group was assessed by MTT assay. **b**, **c** After silencing FMNL2, cell apoptosis in each group was detected by flow cytometry. All experiments were performed three times. The results were shown as mean ± SD. ***p < 0.001 when compared with negative control cells

After silencing FMNL2, proliferation of HGC-27 cells was assessed by MTT assay. As shown in Fig. 3a, the growth of FMNL2 silencing cells was much slower than that of negative control cells (Fig. 3a). These results demonstrate that FMNL2 silencing inhibits proliferation of HGC-27 cells.

Apoptosis plays a crucial role in cell growth. In this study, effect of FMNL2 silencing on apoptosis of HGC-27 cells was assessed by flow cytometry. Results of flow cytometry showed that there was no obvious difference between cells only and negative control cells. FMNL2 silencing cells showed a significant increase in the percentage of apoptotic cells when compared with negative control cells (Fig. 3b, c). These results illustrate that FMNL2 silencing induces apoptosis of HGC-27 cells.

### FMNL2 silencing retards migration and invasion of HGC-27 cells

**Fig. 4:**
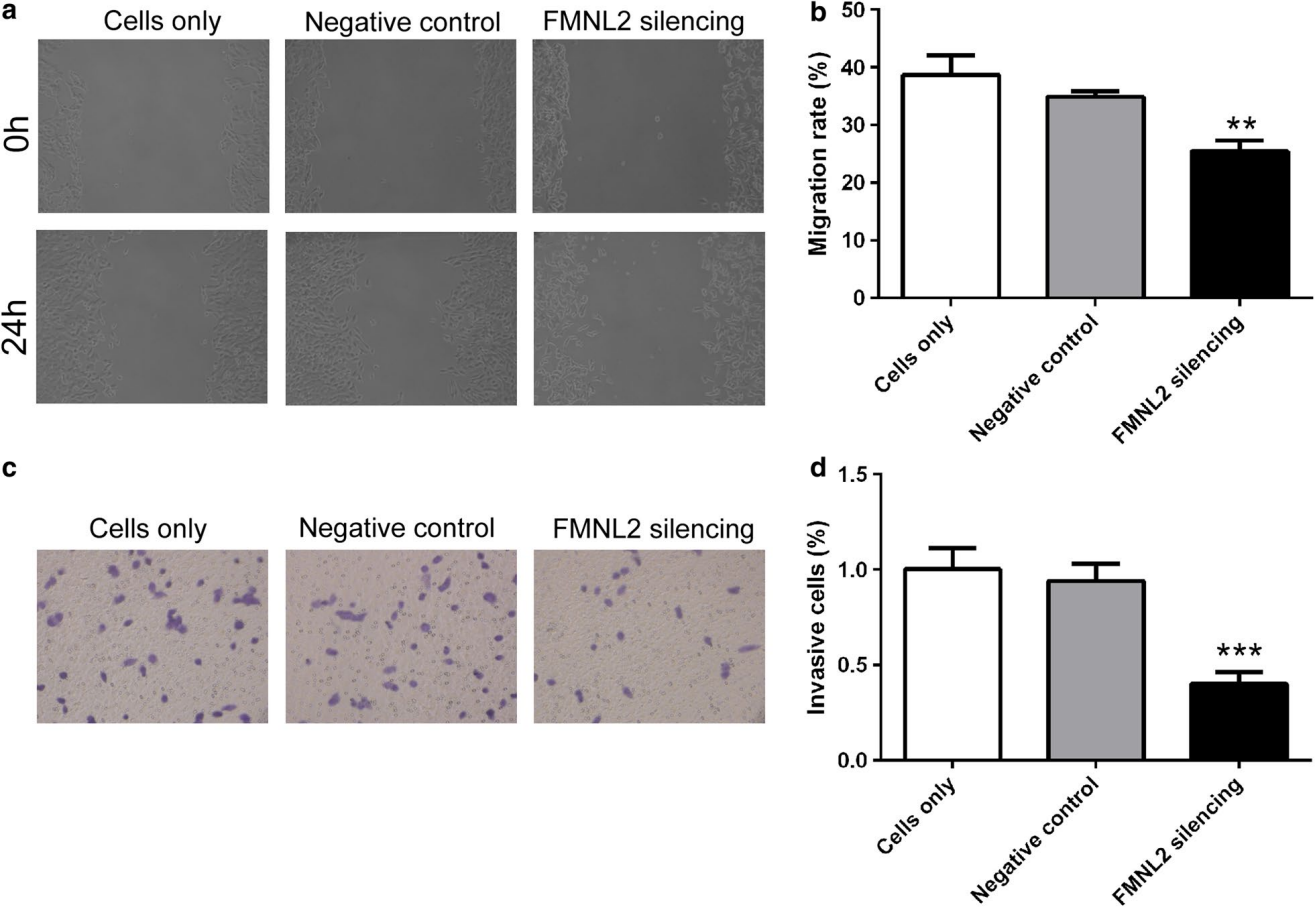
FMNL2 silencing inhibits migration and invasion of HGC-27 cells. **a**, **b** Migration capability of HGC-27 cells in each group was evaluated by wound healing assay. Relative migration rate was calculated. **c**, **d** After silencing FMNL2, invasion capability was assessed by transwell assay. Each experiment was repeated three times. The results were presented as mean ± SD. **p < 0.01, ***p < 0.001 compared with negative control cells

Effect of FMNL2 silencing on migration of gastric cancer cells was evaluated by wound healing assay. Results of our study showed that there was no striking difference between cells only and negative control cells. After silencing FMNL2, the migration rate of FMNL2 silencing cells was 25.45 ± 1.81%, which was significantly declined compared with negative control cells (34.84 ± 0.99%) (Fig. 4a, b). These results show that silencing FMNL2 suppresses migration of HGC-27 cells.

To explore the effect of FMNL2 silencing on invasion of gastric cancer cells, a transwell assay was carried out. As shown in Fig. 4, negative control cells showed no significant difference when compared with that of cells only. While, the percentage of invasive cells of FMNL2 silencing cells was 40 ± 6%, which was decreased significantly compared with that of negative control cells (94 ± 9%) (Fig. 4c, d). These results reveal that silencing FMNL2 suppresses invasion of HGC-27 cells.

**Fig. 5:**
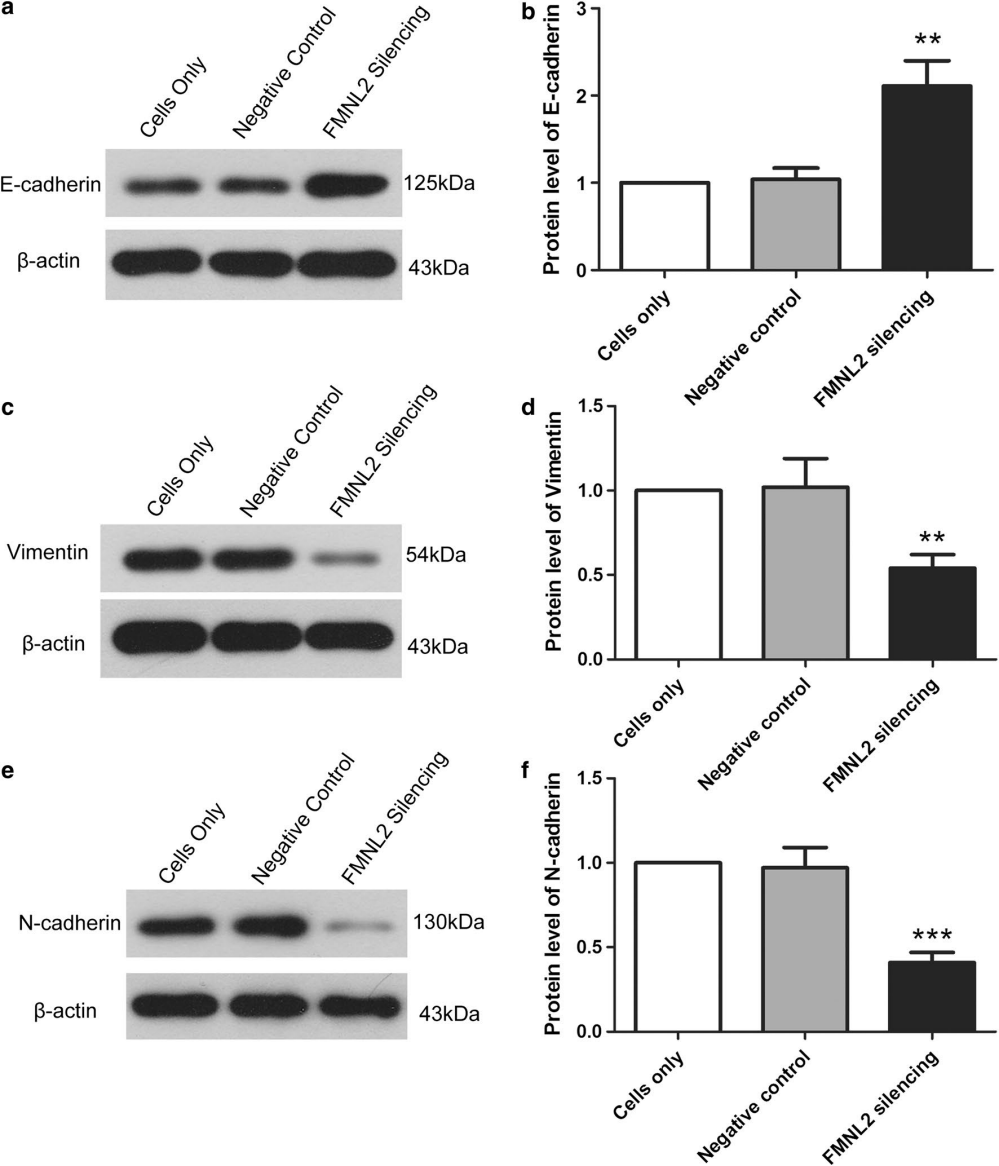
FMNL2 silencing inhibits epithelial-to-mesenchymal transition of HGC-27 cells. **a**, **b** After FMNL2 silencing, protein level of E-cadherin was assessed by western blot. β-actin served as internal control. **c**, **d** Western blot was performed to detect the protein level of Vimentin with β-actin as internal control. **e**, **f** Protein level of N-cadherin was assessed by western blot after silencing FMNL2. Each experiment was repeated three times. The results were shown as mean ± SD. **p < 0.01, ***p < 0.001 compared with negative control cells

Also, proteins associated with epithelial-to-mesenchymal transition (EMT) were also assessed by western blot. After silencing FMNL2, the protein level of E-cadherin, which was an epithelial marker, was increased to 2.11 ± 0.29-fold (Fig. 5a, b). While the levels of Vimentin and N-cadherin, which were mesenchymal markers, were decreased to 54 ± 8 and 41 ± 6%, respectively (Fig. 5c–f). These results indicate that silencing FMNL2 inhibits EMT of HGC-27 cells.

### FMNL2 silencing inhibits integrin internalization induced by PKC

**Fig. 6:**
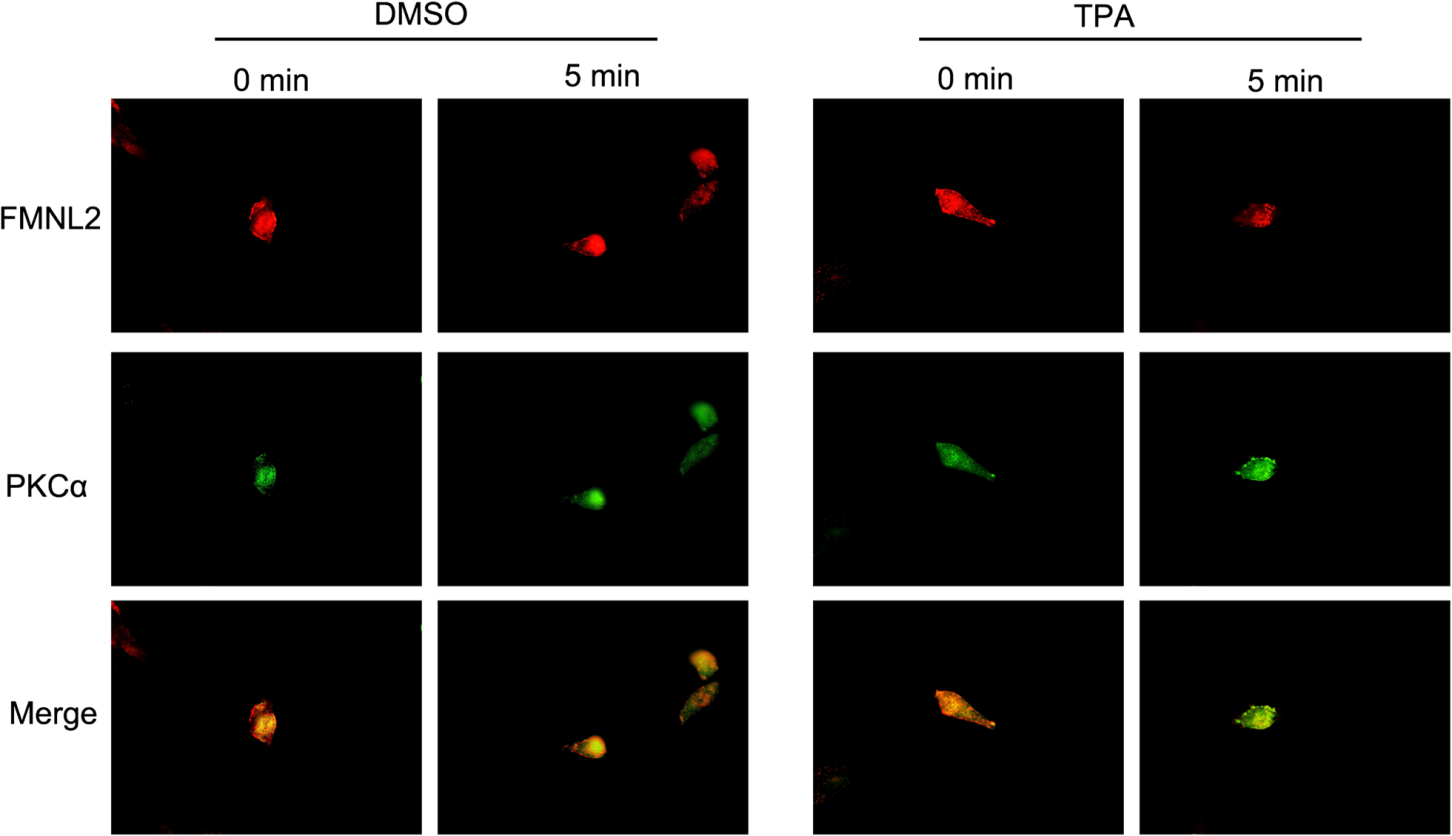
TPA influences the distribution of FMNL2 and PKCα. Before and after TPA or DMSO treatment, the levels and distribution of FMNL2 and PKCα were detected by immunofluorescence. Red fluorescence, FMNL2. Green fluorescence, PKCα

TPA was employed to activate PKC in our study. Upon TPA treatment, more PKCα was concentrated on plasmalemma compared with cells treated with DMSO, indicating the activation of PKC. Moreover, more FMNL2 was concentrated in cytoplasm upon TPA treatment (Fig. 6).

**Fig. 7:**
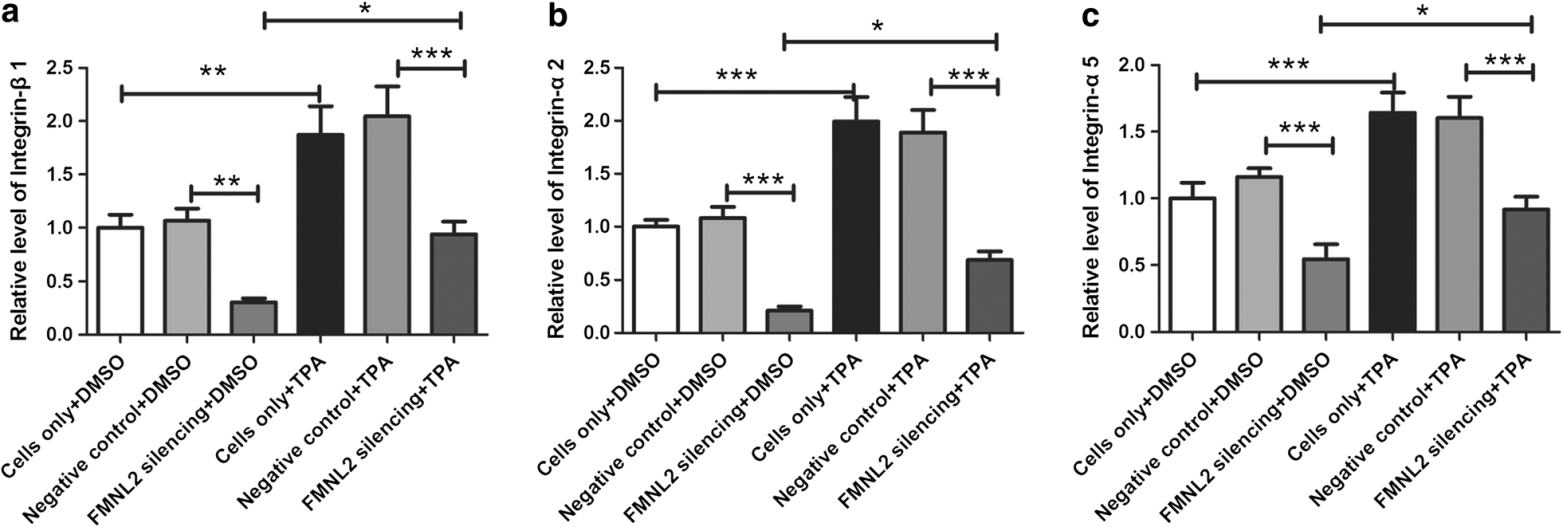
TPA suppresses internalization of integrins induced by PKC. After treatment with TPA, internalization of integrin-β1 (**a**), integrin-α2 (**b**) and integrin-α5 (**c**) was detected. All experiments were repeated three times. The results were presented as mean ± SD. *p < 0.05, **p < 0.01, ***p < 0.001

Additionally, internalization of integrins was also detected in this study. As shown in Fig. 7, activation of PKC by TPA treatment enhanced the internalization of integrin-β1, integrin-α2 and integrin-α5. Compared with negative control cells, silencing FMNL2 significantly decreased the internalization of integrin-β1, integrin-α2 and integrin-α5. In TPA treated cells, silencing FMNL2 also decreased the PKC-induced internalization of integrin-β1, integrin-α2 and integrin-α5. There was no significant difference between FMNL2 silencing + TPA group and cells only + DMSO or negative control + DMSO group. These results demonstrate that silencing FMNL2 reduces PKC-induced internalization of integrins to a nearly normal level.

## Discussion

In the present study, we explored the role of FMNL2 in gastric cancer cells. Silencing FMNL2 suppressed proliferation of gastric cancer cells and induced their apoptosis. FMNL2 silencing also suppressed migration and invasion of gastric cancer cells. Further study showed that internalization of integrins induced by PKC was rescued by FMNL2 silencing. These results of our study indicate that inhibition of integrin internalization may be involved in the effect of FMNL2 silencing on growth and metastasis of gastric cancer cells.

MicroRNAs have close relationships with cancer biology. Clinical trials using microRNA profiling as markers of prognosis and clinical responses were underway [21]. Several microRNAs were reported to suppress growth of colon cancer through targeting FMNL2 [22–24]. Circular RNAs, as emerging biomarkers and targets for cancer [25], recently catch the eyes of researchers. It was reported that circRNA_001569, as a sponge of miR-145, promoted proliferation and invasion of colon cancer through up-regulating FMNL2, which was a functional target of miR-145 [26]. As dysregulation of FMNL2 has been revealed in human cancers and is associated with tumor progression and poor outcomes [19, 20], we speculate that FMNL2 may perform a boosting role in colon cancer growth based on the above indirect clues. And Zhu et al. show that FMNL2 boosts proliferation of colon cancer cells [18]. To our knowledge, only the report of Zhu et al. [18] showed direct evidence for the role of FMNL2 in cancer cell growth. When it comes to gastric cancer cells, there is no direct or indirect evidence. In our study, FMNL2 silencing suppressed proliferation of gastric cancer cells and induced their apoptosis, indicating that FMNL2 may contribute to gastric cancer growth. As far as we know, our study is the first report showing direct evidence for the role of FMNL2 in gastric cancer growth. However, how exactly FMNL2 performs its growth-boosting role in gastric cancer needs to be revealed.

Migration and invasion are crucial initial steps of tumor metastasis. In our study, we also investigated the effect of FMNL2 silencing on migration and invasion of gastric cancer cells. We found that FMNL2 silencing suppressed migration and invasion of gastric cancer cells, indicating that FMNL2 also contributes to metastasis of gastric cancer cells. Consistently, the FMNL2 expression in colon cancer, which has a high FMNL2 level, is correlated with tumor invasion and lymphatic metastasis [16]. FMNL2 is also found to boost invasion and migration of colon cancer [16, 18]. Conversely, in hepatocarcinoma which has a low FMNL2 level, overexpression of FMNL2 suppresses motility and invasion of hepatocarcinoma cells [19]. Literature research shows that FMNL2 has a close relationship with tumor metastasis. First, FMNL2 is a catalyst for polymerization of linear actin. FMNL2 governs many processes, including cytokinesis, morphogenesis, invasion and migration, depended on remodeling of actin [9]. FMNL2 accumulating at lamellipodia and filopodia tips contributes to actin filament nucleation and elongation, and boosts actin assembly [27, 28]. Second, FMNL2 also modulates EMT which is an important phenomenon contributing to tumor metastasis. The expression of FMNL2 is negatively correlated with epithelial marker E-cadherin and positively correlated with Vimentin. And knockdown FMNL2 leads to EMT, with elevated E-cadherin and declined Vimentin and Snail [17]. Lossing FMNL2 also decreases TGF-β-induced EMT [17]. In this study, we also confirmed a similar effect of FMNL2 on the protein levels of EMT markers in gastric cancer cells.

The integrin signal has a close relationship with tumor progression, including cell proliferation, migration, invasion and differentiation. Integrins interact with extracellular matrix to provide traction which is required for tumor cell invasion [8]. Integrins also contribute to tumor invasion through regulating matrix metalloproteases (MMPs) which are critical to the proteolysis of matrix proteins [8, 29]. The ability of cell proliferation is depending on collagenous matrix status [30]. Through regulating MMPs and collagenous matrix status, integrins may also influence tumor cell proliferation. Moreover, integrins are revealed to control the expression of cyclins and cyclin-dependent kinase inhibitors [31, 32], which are key regulators of cell cycle progress, thus contributing to cancer cell proliferation. Integrins are also implicated in the process of EMT [33, 34], a critical process triggered during tumor metastasis, and contribute to tumor angiogenesis and chemoresistance [30].

Interestingly, integrin trafficking (internalization and recycling) is very important in controlling integrin actions [3]. Tissue factors, such as epidermal growth factor (EGF), platelet-derived growth factor (PDGF) and protein kinase C (PKC), have been shown to induce integrin internalization [35–37]. Meanwhile, dysregulation of integrin trafficking is implied in tumorigenesis [38], and integrin internalization is reported to boost cell migration [3]. Due to the important role of integrin internalization, targeting integrin trafficking is regarded as a potential cancer therapy [38]. FMNL2 is required for integrin internalization downstream of PKC. PKC phosphorylates and activates FMNL2, leading to direct binding of FMNL2 to cytoplasmic tails of integrin-α for integrin-β1 endocytosis [3, 39]. Wang et al. also showed that FMNL2 promoted integrin trafficking downstream of PKC [39]. Consistently, in our study, FMNL2 silencing declined integrin internalization induced by PKC in gastric cancer cells. We speculate that integrin internalization may be implicated in the role of FMNL2 in gastric cancer cells.

## Conclusion

Our study demonstrates for the first time that silencing FMNL2 suppresses proliferation, invasion and migration and induced apoptosis of gastric cancer cells. Additionally, integrin internalization may be implicated in the role of FMNL2 in gastric cancer cells. Our study indicates that FMNL2 may become a potential therapeutic target for gastric cancer.

## Abbreviations

FMNL2: Formin-like 2
PKC: protein kinase C
GTPase: guanosine triphosphatase
DRF: diaphanous-related Formin
qRT-PCR: quantitative real-time PCR
TBST: Tris buffered saline with Tween
HRP: horseradish peroxidase
MTT: 3-(4,5-dimethyl-2-thiazolyl)-2,5-diphenyl-2-H-tetrazolium bromide
PBS: phosphate buffered saline
FITC: fluorescein isothiocyanate
PI: propidium iodide
TPA: 12-*O*-tetradecanoylphorbol-13-acetate
ELISA: enzyme-linked immunosorbent assay
EMT: epithelial-to-mesenchymal transition
MMP: matrix metalloprotease
EGF: epidermal growth factor
PDGF: platelet-derived growth factor
